# JinQi-Jiangtang tablet, a Chinese patent medicine, for pre-diabetes: a randomized controlled trial

**DOI:** 10.1186/1745-6215-11-27

**Published:** 2010-03-10

**Authors:** Hongbo Cao, Ming Ren, Liping Guo, Hongcai Shang, Junhua Zhang, Yuzhen Song, Hui Wang, Baohe Wang, Xiantao Li, Jing Hu, Xuemei Wang, Dehui Wang, Jianzong Chen, Shuanglei Li, Liming Chen

**Affiliations:** 1 Clinical Assessment Institute, Tianjin University of TCM, Yuquan street, Tianjin, 300193, China; 2 Baokang hospital, Tianjin university of TCM, Yuquan street, Tanjin, 300193, China; 3 Evidence Based Medicine Centre of Tianjin, Anshanxidao street, Tianjin, 300193, China; 4 Department of Integrated Chinese and Western Medicine, The First Hospital of Peking University, Xishiku Street, Xichen district, Beijing, 100034, China; 5 Department of Endocrinology, The Second Affiliated Hospital of Tianjin University of TCM, Zhenli Street, Hebei District, Tianjin, 300150, China; 6 Department of TCM, Xijing hospital, Changlexilu Street, Xi'an, 710032, China; 7 Department of Endocrinology, The First Affiliated Hospital of Guangxi College of TCM, Yuanhu Street, Nanning, 530023, China; 8 Department of Endocrinology, Metabolic Disease Hospital of Tianjin Medical University, Pingjiang Street, Hexi District, Tianjin, 300211, China

## Abstract

**Background:**

Pre-diabetes is a growing health concern where a large percentage of these patients develop full type 2 diabetes. Effective interventions on pre-diabetes can prevent or delay the occurrence or development of diabetes. Pharmaco-dynamics and pre-clinical of JinQi-Jiangtang tablets (JQJT) suggest that it could be benefit for pre-diabetes.

**Methods/Design:**

Randomized controlled trial (RCT) is implemented in this study. The study term is 24 months (12 months for intervention and 12 months for follow up). Participants are recruited from four cities of China: Beijing, Tianjin, Xi'an and Nanning. Four hundred participants are randomized to treatment group (JQJT tablets) and control group (Placebo); two hundred participants each. People being included in this study must have been diagnosed as pre-diabetes via western medicine criteria and traditional Chinese medicine (TCM) criteria. The end-point indexes include: incidence of diabetes mellitus and reversion rate. Primary outcome indexes include: oral glucose tolerance test; insulin releasing test; glycosylated hemoglobin (HA1c). Secondary outcome indexes include: score of the Short Form 36 Health Survey Questionnaire (SF-36); score of TCM symptoms; blood lipid test. Indexes of safety include: general medical examination; blood and urine regular test; electrocardiogram (ECG), liver function (ALT) and renal function (BUN, Creatinine) test; record of adverse event, such as headache, faint, etc. Qualitative control will be implemented and a number of standard operating processes (SOPs) will be formed throughout the study: laboratory quality control measures; compliance control for researchers and participants; researcher training before study; supervision; investigational drug management and others.

**Discussion:**

The aim of this study is to evaluate the effectiveness and safety of JinQi JiangTang (JQJT) tablets for the treatment of patients with pre-diabetes.

**Trial registration:**

Chinese clinical trials register ChiCTR-TRC-00000401

## Background

An increasing number of people experience complications from symptoms of pre-diabetes. Some studies have discovered that about 5%-10% of pre-diabetes will develop into diabetes within one year and the occurrence of cardiovascular events in patients with pre-diabetes are higher than in healthy individual. According to Hoorn[1], the rates of development of diabetes after 5.8-6.5 years follow-up from patients with normal glucose tolerance, single impaired fasting glucose (IFG), single Impaired Glucose Tolerance (IGT), both IGT and IFG people are 4.5%, 33.0%, 33.8% and 64.5%, respectively. Therefore, the prevention of type 2 diabetes in pre-diabetic patients is a topic of importance in diabetes research.

### Progress of pre-diabetes

Effective interventions on pre-diabetes can prevent or delay the occurrence or development of diabetes; it can also reduce micro-vascular and macro-vascular complications. The result of the Daqing study from 1986-1994 concluded that lifestyle interventions could reduce the incidence of diabetes by 30%-50%[2]. However, another study confirmed that lifestyle interventions were not completely effective to prevent the development of type 2 diabetes for high-risk groups[3]. Another study showed a high trend of body weight rebound and blood glucose increase after 1-2 years of lifestyle interventions [4]. STOP-NIDDM discovered that IGT intervention was able to reduce the occurrence of diabetes by 36% [5]. DPP discovered that metformin and troglitazone can significantly reduce the risk of diabetes [6]. In the TRIPOD study, troglitazone reduced the risk of diabetes by 55% in gestational diabetes patients [7]. The latest DREAM trial showed that the intervention of pre-diabetes can delay the disease process [8,9]. These studies give us hope to prevent diabetes effectively.

### Prevention and treatment on pre-diabetes in traditional Chinese medicine (TCM)

Traditional Chinese medicine (TCM) can treat pre-diabetes effectively[10]. A systematic evaluation of pre-diabetes intervention, including 19 randomized controlled trials, showed that the combination of Chinese medicine and lifestyle intervention can decrease fasting blood glucose in 1471 cases. Meanwhile, the 2 hours postprandial blood glucose and 2 hours postprandial insulin levels were lower than a simple lifestyle intervention [11]. Zhou Zhuoning[12]considered that diabetes can be explained by three TCM Syndrome from IGT to diabetes as: yin deficiency with heat, dual deficiency of yin and qi, dual deficiency of yin and yang in development.

### Constituents of JQJT tablets

Alkaloid is the major constituent of Coptis, including Berberine, palmatine, methyl and coptisine. Berberine is the most content among them (accounting for 6.88%~13.64%). The main chemical constituents of Astragalus is flavonoids, saponins and polysaccharides. The main chemical composition of Honeysuckle is volatile oils, flavonoids, organic acids and other substances, chlorogenic acids compound is the main active ingredient among them. Berberine, flavonoids can lower blood sugar significantly and chlorogenic acids compounds can strengthen the immune system.

### Pharmaco-dynamics of JinQi-Jiangtang(JQJT) tablets

• JinQi-Jiangtang (JQJT) tablets is composed by Coptis, Astragalus, and Honeysuckle.

• Influence on glycometabolism: Studies before proved that JQJT tablets can improve mice glucose tolerance obviously[13]. Honeysuckle can decrease blood sugar[14]. Berberine, as a natural plant alkaloid isolated from the Coptis chinens, has been noted for its potential glucose lowering effect[15].

• Influence on insulin resistance(IR) and serum insulin: JQJT tablets can improve the insulin resistance caused by Hydrocortisone and decrease the serum insulin in mice[16].

• Influence on immune function: JQJT tablets can improve immune function and protect thymus gland atrophy of mice [16].

### Pre-clinical of JQJT tablets

Sheng zhufang proved that JQJT tablets can improve insulin resistance in humans[17]. Gu wenyuan indicated that combination of JQJT tablets and Gliclazide can improve fasting blood-glucose (FBG) of 2 type diabetes patients[18]. Zhang rongrong discovered that JQJT tablets can not only prevent the progression from pre-diabetes to diabetes but also delay development of diabetic nephropathy (DN)[19].

## Methods/Study design

### Objectives

To evaluate the effectiveness and safety of JQJT tablets for pre-diabetes in China by comparison with placebo.

### Research type

Randomized controlled trial (RCT).

### Screening of participants

400 pre-diabetes patients will be divided into the treatment group and the control group (1:1). Participants should be included according to diagnosis criteria and inclusion criteria (reference to the next section).

### Intervention

• Arrangement for intervention: All researchers in this study will be trained before their participation. Some measures will be implemented in order to ensure compliance, such as compensation and prize. Patient handouts will be delivered to every participants to instruct their medication and cooperation.

• Lifestyle intervention: Researchers will develop an individual diet program for each participant, including reasonable match of calories, protein and carbohydrates, low-salt and low-sugar diet, advise them to give up some bad diet habits. Risk factors, such as smoking and alcoholics, will be controlled strictly. Individual's exercise program will be formulated for all participants according to their age, gender, etc.

• Drug intervention: The period of drug intervention is 12 months. All participants have to visit researchers every month. The interventions are as follows: JQJT tablet for treatment group, taking seven tablets each dose and twice daily, all the drugs are taken before meal; Placebo of JQJT for control group, the method and time of taking drugs is the same as treatment group.

### Randomization

The randomization of the trial will be completed by the independent data center named Interact Voice Responding System (IVRS). According to a random sequence table (generated by SAS8.2), participants who satisfied the inclusion criteria will be allocated randomly into one of the two groups with a ratio of 1:1. The identification code and random number, which is unique for each participants, will be generated by the IVRS.

### Allocation concealment

All researchers will receive training of allocation concealment at the central randomization office. The randomization sequence schedule is held and controlled by the appointed one who is not involved in this trials. The schedule is secret for researchers, participants and other research personnel before study, so allocation sequence is implemented in a secure manner to prevent foreknowledge by either of them.

### Blindness

Double-blinding method is adopted in this study. Simulated agents of JQJT tablet have the same appearance, shape, color and packaging with JQJT tablets, so researchers and participants can not know the kind of medication and group.

Twice unblinding method is adopted in this study. Firstly, after close of data-base, the one who keep the blind code will deliver a allocation schedule only signed with group A and B to statistics department. Secondly, after the handing of statistical outcome and final report, the one keeping the blind code will declare real meaning of group A and B. In case of emergency during this trials, the blinding will be broken after obtaining the consent of principle investigator.

### Sample size

According to the results of the "JQJT tablets intervene IGT study" by Professional Committee of Diabetes, Association of Integrative Medicine in Tianjin, annual average tyoe 2 diabetes conversion rate of test group is 4.05%, control group is 12.69%. The sample was estimated according to the parameters: *α *= 0.05, *β *= 0.1

Considering attrition of no more than 20%, the total number of qualified cases in the groups will be 200 each (n = 400).

#### Patient identification and enrollment

**Diagnosis criteria **(Guidelines for Clinical Diagnosis and Treatment of Diabetes in 2008, ADA)

Impaired fasting glucose (IFG) (FPG5.6-6.9 mmol/L) &/or impaired glucose tolerance (IGT) (FPG <5.6 mmol/L & OGTT 2 hPG 7.8-11.1 mmol/L)

**TCM symptoms **(Chinese New Medicine Treatment of Diabetes Clinical Research Guiding Principle)

Dual deficiency of qi and yin or yin deficiency with heat.

##### Inclusion criteria

• Fulfill pre-diabetes diagnostic criteria;

• Fulfill TCM diagnostic criteria;

• Aged 18-75;

• Completed and submitted informed consent form;

##### Exclusion criteria

• History of diabetes (except gestational diabetes);

• Cardiovascular events(cerebrovascular accident in 6 months, history of myocardial infarction or heart failure; severe organic heart disease; aneurysm with the main artery or dissecting aneurysm; specific angina, type II degree atrioventricular block, sick sinus syndrome);

• Impaired hepatic and renal function: AST and/or ALT 2- fold the upper limit of normal or above; same for creatinine. Urine protein > ++ and/or hematuria.

• Fasting triglycerides ≥ 10 mmol/L;

• Endocrine disease, such as hyperthyroidism, autoallergic disease, cancer or other serious fatal illness;

• Former use of glucocorticoid, β receptor blockers, thiazide diuretics and nicotinic acid;

• Pregnancy, preparation of pregnancy and lactating women;

• Suffering from mental diseases or non-cooperation patients;

• Participating in other clinical trial within the last two weeks;

• People refusing to provide consent for the study.

##### Rejection criteria

• Could not take medicine according to the protocol.

• Data is incomplete.

##### Suspension criteria

• Poor compliance.

• Serious adverse events, complications and special physiological changes.

• Unblinded abnormally.

• Reluctance to continue this study.

• Taking medicine forbidding in this study.

• Withdraw for various reasons, such as failure to follow up or death.

• Incomplete information which will influence the study.

• Big mistake in protocol or significant deviation in implement (such as the leak of blind).

• National laws, ministry of science and technology or other authorities decided to terminate the study.

#### Follow up

##### Points of data capture (figure 1)

**Figure 1:**
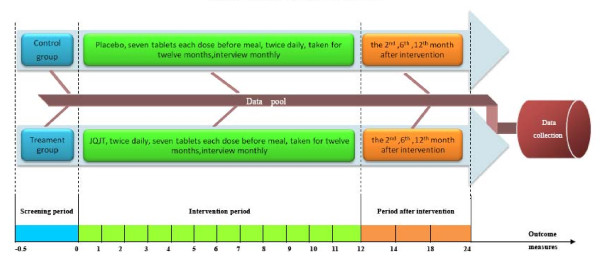
**Points of data capture(unit: month)**.

• Intervention period: 12 months; once a month for 12 months

• After intervention: the 2nd, 6th, 12th month after intervention

##### Contents

Data should be captured according table 1 and 2.

**Table 1:** Content of data capture(to be continued)

Item	Screening period (unit: month)	Intervention period (unit: month)											
	
	-0.5	1	2	3	4	5	6	7	8	9	10	11	12
ICF	X												

Record of medical history	X												

Demographic information	X												

General physical examination	X	X	X	X	X	X	X	X	X	X	X	X	X

FBG	X	X	X	X	X	X	X	X	X	X	X	X	X

PBG	X	X	X	X	X	X	X	X	X	X	X	X	X

OGTT	X	X	X	X	X	X	X	X	X	X	X	X	X

Insulin release test	X												X

HbA1c	X												X

serum TC, LDL-C, VLDL-C	X												X

Blood and urine regulation	X						X						X

ALT, AST	X			X			X			X			X

BUN, Cr	X			X			X			X			X

Electrocardiogram	X												

Pregnancy Test	X						X						X

Selection/exclusion criteria	X												

Drugs of prohibition	X												

SF-36	X												X

Evaluation of TCM Syndrome	X												X

Obtain random code	X												

Dial 800 to Assign drug	X			X			X			X			

Drug Distribution and record	X	X	X	X	X	X	X	X	X	X	X	X	

End-point events		X	X	X	X	X	X	X	X	X	X	X	X

Adverse events	X	X	X	X	X	X	X	X	X	X	X	X	X

Drug combination		X	X	X	X	X	X	X	X	X	X	X	X

X: Item need to be done during different period.

**Table 2:** Content of data capture

Item	Period after intervention (unit: month)		
	
	14	18	24
General physical examination	X	X	X

FBG	X	X	X

PBG	X	X	X

OGTT	X	X	X

Insulin release test			X

HbA1c			X

Serum TC, LDL-C, VLDL-C			X

ECG			X

Blood and urine regulation			X

ALT, AST, BUN			X

SF-36			X

Evaluation of TCM Syndrome			X

Pregnancy test		X	X

End-point events	X	X	X

Adverse events	X	X	X

Drug combination	X	X	X

X: Item need to be done during different period.

#### Outcome measures

##### Index of end point

• Occurrence of type 2 diabetes (according to the guideline of ADA 2008);

• Blood glucose change to be normal.(FPG<5.6 mmol/L & OGTT 2 hPG <7.8 mmol/L)

##### Primary outcomes

• Oral glucose tolerance test

• Insulin releasing test

• Glycosylated hemoglobin (HA1c)

##### Secondary outcome measures

• Score of the Short Form 36 Health Survey Questionnaire(SF-36)

• Scoring of TCM symptom (refer to clinical research guidance of new Investigational drug in TCM)

• Blood lipid: Total Cholesterol (TC), Triglyceride(TG), High Density Lipoprotein-Cholesterol(HDL-C), Low Density Lipoprotein-Cholesterol(LDL-C)

##### Safety index

• General medical examination

• Study visit blood and urine tests

• Functional examination: EKG, ALT, BUN, Cr

• Adverse events.

#### Measure and evaluate tools

***Evaluation criteria of efficacy on diabetes conversion rate ***(refer to the guideline of the diagnosis of diabetes mellitus of ADA 2008)

***The rate of blood glucose return to normal ***(refer to the guideline of the diagnosis of diabetes mellitus of ADA 2008)

##### The score of SF-36

Questionnaire is the evaluation of various dimensions, entries, and between groups and within group comparison of questionnaire total score. The score is calculated with measurement data.

***Evaluation of TCM symptom score ***(refer to clinical research guidance of new Investigational drug in TCM)

Quantitative criteria of TCM symptom grades.

#### Adverse Event Monitoring

##### Report of adverse event

When adverse event happens, researchers must fill out the "SAE form" and report it to project office and center ethics committee immediately. The event should also be reported to the safety supervision division of the State Food and Drug Administration within 24 hours.

##### Record of adverse event

Adverse event report form must be filled according to the real circumstances. Some information, including occurrence time, severity, duration, adopted measure of the adverse event should be noted as well.

##### Relationship between adverse event and investigational drugs

Judgment of the relationship between the administration time and adverse reactions; judgment of the relationship between suspected and known adverse reactions of investigational drug; whether suspected adverse reactions disappear or mitigate after discontinuation; whether the same reaction occurred again after taking investigational drug once more.

##### Security evaluation criterion

• Safe without any adverse drug reaction.

• Relative safe with mild adverse drug reactions and unnecessary treatment.

• Having moderate adverse drug reactions and necessary treatment.

• Stopping to take investigational drugs due to adverse reactions.

#### Statistical Analysis Plan

##### Analysis parameters

All parameters will be analyzed by SAS 8.2 software package.

##### Analysis of data sets

• Full analysis set: According to the principle of analysis for intention to treat (ITT), participants will be rejected by smallest and reasonable method. The last observation carried forward (LOCF) method will be used for the missing data supplement.

• Per-protocol set (PPS): PPS should be used in the patients who satisfied with the following characters: compliance is between 80% and 120%; not using prohibited drugs; fit with inclusion criteria; case of completed observation.

• Safety set (SS): All information of safety record will be assessed, including adverse events and laboratory test results.

• Statistical analysis method: Relative risk reduction (RRR), absolute risk reduction (ARR), number needed to treat (NNT) adopt nonparametric methods to evaluate the effectiveness. To adopt t-test, repeated measure analysis of variance, radit test, rank test, survival analysis, Cox proportional hazards regression model and so on.

##### Mathematical statistics analysis and expression

• Comparison between groups: The chi-square test for enumeration data; Wilcoxon rank sum test for non-normal distribution of measurement data; t-test for normal distribution of measurement data; ANOVA for repeated measurement data; Ridit test for ranked data; CMH test for the center effect.

• Dropping analysis: The chi-square test, or Fisher exact probability method.

• Compliance analysis: The chi-square test, or Fisher exact probability method.

#### Documents conservation and summary

The documents such as informed consent, signature of participants and other cases are requested to conserve clearly according to GCP by every unit after the study. Researchers should preserve the material of clinical trial for five years.

#### Data management

##### Data input

Database manager designs EpiData3.1 database. The data manager carries on the test to the database base on analogue data or real CRF data. Data are inputted by two full time personnel independently.

##### Data verification

• Comparing with duplicate input result.

• Checking data uniformity and logic.

• Spot-checking 10% CRF, compare database with CRF form.

##### Data locked-up

The main data will be locked up until research completed. New question will carried on statistical analysis procedure after data locking; all contents and the revision should be preserved well. For the questions in the CRF, data manager should produce DQF and inquire the researchers through clinical supervisor. Data manager should make data modification, affirmation and record according to the reply of researchers.

#### Management of investigational drug

JQJT tablets and placebo are all supplied by Longshunrong Pharmaceutical Company in Tianjin. According to double-blind method, the packing of placebo should be consistent with the investigational drug. Researchers will be provided with the drug serial number and distribute drugs to participants monthly through 800 telephone terminals. The drug managers take charge of preservation of investigation agent. When adverse event happens, researchers can uncover blindness through the DSMB.

#### Trial management

##### Change of protocol

All changes of protocol should be preserved and any modification of the protocol, including ICF, must get the approval of ethics committee.

##### CRF tracking

All signed ICF must be handed over even if some of them are unqualified; any questions of CRF or comments must be submitted directly to the organizer.

##### Change of research center

The decision to change research center should be careful and these change must be reported to ethics committee. Research center would be changed for slow speed of study, severe violation of protocol and GCP.

#### Researcher's responsibility

Researcher must understand protocol details and adhere to it strictly. They should understand the related information of investigation agent, including function, curative effect and security. They should have enough time to complete this study on schedule and have responsibility to explain study process and consent form to participants. Participants with adverse events should be treated suitably and related data should be recorded accurately and completely in primitive medical record and CRF. Researcher should accept censorship or examination from organizer of clinical trial or other inspected departments.

#### Quality control

##### Quality control of laboratory

• All hospitals should establish uniform SOP and quality control sequence.

• All units should offer researcher suitable medical equipments and emergency facilities.

• Certain item should be in charged by special personal.

##### Request for researcher

Researchers in this study must possess the qualifications and ability to carry on it and they should not be changed constantly.

##### Measures for compliance of participants

Researchers should give investigational drug to in-patient in time and explain medication method to outpatient. Compliance of participants will be inspected by method of drug notation. They are asked to bring drug last time in order to calculate the number.

##### Monitoring and inspection

Monitor should constantly visit each unit to know the situation of facility and research follow, reexamine CRF, check storage of investigational drug and record of data.

According to GCP, organizer will carry on conventional and emphasis inspection on related activities and documents of every unit during study in order to evaluate whether the process of study is in accord with protocol and standard practice, whether collected data are recorded prompt, accurate and completely.

##### Bias control

Any other west or TCM drugs which has similar effects as the investigated agent will be prohibited when participants decide to participate this study. Researcher should pay close attention to contamination and disturbance in this study in order to prevent occurrence of bias.

##### Ethics

China clinical trial quality management norm/Helsinki declaration

The study will guarantee to abide Chinese clinical trial quality control standard and Helsinki manifesto.

#### Ethics committee

Before the beginning of study, all hospitals should provide related data about qualification of the main researcher and laboratory conditions in order to help ethics committee decide their participation. Each unit needs not appraisal ethics again unless special request and they can contract ethics committee and state reasons if necessary.

#### Inform consent form

ICF must be reviewed and approved by ethics committees before study. Researchers should inform participants' relevant information in words and writing meanwhile by understandable language. ICF must be signed by the participants or their representatives with date. Before the signature of ICF, participants and representatives should have adequate time to read it. Signed ICF should be preserved properly by researchers and participants independently.

#### Security

Researchers should keep related information of participants strictly in this study. Capital forms, numbers or codes can be used to identification rather than names of participants in CRF or other documents. Researchers should preserve selected table which record code, name and home address of participants.

## Abbreviations

[JQJT]: Jin qi jiang tang; [CRF]: Case Report Form; [ICF]: Inform consent form; [GCP]: Guild contribution point; [CRO]: Contract Research Organizations; [RRR]: Relative risk reduction, [ARR]: Absolute risk reduction; [NNT]: Number needed to treat; [SS]: Safety set; [PPS]: Per-protocol Set; [ITT]: Intention to treat; [SAE]: Serious adverse event; [TC]: Total cholesterol; [HDL-C]: High density lipoprotein cholesterol; [LDL-C]: low density lipoprotein cholesterol; [TG]: Triglyceride; [ECG]: Electrocardiogralph; [OGTT]: Oral glucose tolerance test; [HbA1c]: Hemoglobin A1c; [IR]: Insulin releasing; [ALT]: Alanine transaminase; [AST]: Aspartate aminotransferase; [BUN]: Urea nitrogen; [Cr]: Creatinine; [IFG]: Impaired fasting glucose; [IGT]: Impaired glucose tolerance; [IVRS]: Interactive Voice Response System; [RCT]: Randomized controlled clinical; [SF-36]: Short Form 36 Health Survey Questionnaire

## Competing interests

The authors declare that they have no competing interests.

## Authors' contributions

HS, LG designed and finalised the protocol; HC, MR, YS were in charge of the trial management. LX, BW were in charge of estimate of sample size and statistics of result. JZ, HW, JH were in charge of monitoring. HS particularly developed this publication describing the trial protocol and endpoint assessment. WX, WD, CJ, LS, CL will assist with recruitment of participants. All the authors read and approved the final manuscript.
